# Lanthanide-containing visible-light-excited thermosensitive luminescent films

**DOI:** 10.3389/fchem.2026.1786361

**Published:** 2026-03-25

**Authors:** Aleksandr S. Krupin, Andrey A. Knyazev, Ruzanna M. Ziyatdinova, Yuri G. Galyametdinov

**Affiliations:** Physical and Colloid Chemistry Department, Kazan National Research Technological University, Kazan, Russia

**Keywords:** aggregates, lanthanide(III) complexes, luminescent sensors, thermal sensitivity, visible-light-excited luminescence

## Abstract

Among the methods of temperature measurement, luminescent thermometry has recently become increasingly interesting due to a number of advantages over traditional thermometers such as high sensitivity and spatial resolution, no need for contact with the analyzed object, and the capability for remote monitoring of temperature fields in complex conditions. Despite the obvious advantages, the practical application such sensors are currently limited by the following problems: low photo- and thermal stability, the use of UV sources for their excitation and the difficulty of obtaining thin-film materials. In this work, we propose a simple approach to developing visible-light-excited temperature sensors based on films of an anisometric Eu(III) complex by performing their vitrification from the melt. Due to formation of J-aggregates, the spectra of such microscale films contain an intensive excitation band at ∼400 nm, which allows for activating their luminescence not only by UV light but also by inexpensive visible light sources with the absorption range wavelengths of 390–425 nm. The impact of temperature on the luminescence intensity and lifetime of the films excited by visible light was studied. The ranges of temperature measurement and sensitivities of the produced films were evaluated. The films demonstrated high temperature sensitivity of their luminescence equal to 18.97 μs/K. The produced materials can reversibly change their luminescent parameters in the temperature range of 298–353 K. The studied temperature-sensitive material can be excited by inexpensive sources of visible light. Such an approach is promising for enhancing the photostability of sensing elements and reducing the overall cost of temperature measurement devices.

## 1 Introduction

Temperature is one fundamental thermodynamic parameter, which characterizes the state of a physical system. It plays a crucial role in monitoring and optimizing the majority of industrial, medical, environmental, and research processes (Allison and Gillies, 1997; Childs et al., 2000; Jaque et al., 2014; Moldover et al., 2016; Okabe et al., 2012; Rodríguez-Sevilla et al., 2022). Modern approaches to measuring temperature include liquid thermometers, thermocouples, resistive sensors, and pyrometers. However, traditional methods often require a physical contact with the object, which limits their remote monitoring capabilities. Pyrometers, although allowing contactless measurement, show poor accuracy and offer low spatial resolution, particularly for measuring temperatures inside macroscale objects (Allison and Gillies, 1997; Brites et al., 2016; Brites et al., 2023; Childs et al., 2000; Moldover et al., 2016).

The problems of traditional temperature measurement methods are effectively solved by luminescent thermometry, which allows for remote and noninvasive measurement of temperature at microscale and nanoscale by analyzing changes in luminescent characteristics with respect to temperature (Brites et al., 2023; Dramićanin, 2020; Dramićanin, 2020; Goderski et al., 2020; Goderski et al., 2020; Jaque and Vetrone, 2012; Jaque and Vetrone, 2012; Quintanilla et al., 2015; Quintanilla et al., 2015; Runowski et al., 2018; Runowski et al., 2018; Đačanin Far and Dramićanin, 2023; Đačanin Far and Dramićanin, 2023). This method is based on the behavior of the luminescence intensity or lifetime, or spectral line positions calibrated relative to temperature. Each approach has its advantages and disadvantages, including sensitivity to environmental conditions and challenging instrumental implementation (Brites et al., 2023; Choi et al., 2014; Maturi et al., 2021; Zheng et al., 2021). Luminescent thermometry is actively developing due to its unique advantages over traditional contact methods such as high sensitivity and spatial resolution, no need for contact with the analyzed object, and the capability for remote monitoring of temperature fields in complex conditions.

Typical phosphors for luminescent thermometry are represented by organic dyes, BODIPY, semiconductor nanoparticles, MOF, lanthanide compounds, etc. (Brites et al., 2012; Brites et al., 2019; Brites et al., 2023; de Oliveira Lima et al., 2021; Jahanbazi and Mao, 2021; Martins et al., 2022; Yamazaki et al., 2023; Đačanin Far and Dramićanin, 2023). Ln (III) compounds are becoming increasingly popular for their unique spectroscopic properties such as monochromatic luminescence, high quantum yields, large Stokes shifts, and long luminescence lifetime. These properties arise from the electronic transitions within the f-shells of Ln (III) atoms. Complexes of these metals demonstrate sufficient stability, resistance to photodegradation, and a wide range of temperature-sensitive optical responses. Most Ln (III) compounds exhibit their luminescent properties after excitation by UV light. However, a significant limitation to practical applications of UV light sources is their low brightness and high cost. In this respect, a promising approach is development of Ln (III) compounds excitable in the visible light range. Excitation spectra can be changed by varying concentration and controlling the aggregation state of phosphors. Therefore, the efficient use of phosphors in various devices requires consideration of their luminescence behavior in solutions and films. The studies of the impact of aggregation on luminescence performed for BODIPY organic dyes, their analogues and homologues (Antina et al., 2021; Binnemans, 2005; Fedorenko et al., 2014; Gemen et al., 2020) revealed that an increase in the concentration of a phosphor in solutions leads to changes in the absorption and excitation spectra due to formation of two types of aggregates. For the J-aggregates, a shift of absorption and/or excitation spectra to a longer wave area was observed. For the H-aggregates, a shift to a shorter wave area was detected (Bricks et al., 2017; Deshmukh et al., 2022; Li et al., 2021; Xu et al., 2021). Thus, it is possible to perform a targeted modification of the luminescent properties of such materials in their solutions and films. Formation of the J-aggregates can be used for developing visible-light-excited luminescent materials. However, the review of literature reveals only limited examples of temperature sensitive materials based on Ln (III) complexes, which can be excited by visible light (Berry et al., 1996; Borisov and Klimant, 2008; 2012; Borisov and Wolfbeis, 2006; Kozak et al., 2014; Li et al., 2016; Ohmagari et al., 2022).

In our previous works, we demonstrated that the powders of anisometric Eu(III) complexes are amorphous. They virtually do not crystallize and possess high softening temperatures. Therefore, such complexes can be used for producing transparent vitrified films (Knyazev et al., 2022a; Lapaev et al., 2020; 2023). The presence of aggregates in such vitrified films allows for using visible light sources for their excitation (Knyazev et al., 2025). This work aims at studying the impact of supramolecular organization on the optical properties and thermal sensitivity of the luminescence of the films based on anisometric Ln (III) compounds. Formation of the J-aggregates in microscale vitrified films was shown to provide them with capabilities of excitation by not only the UV light but also by inexpensive visible light sources with the wavelengths corresponding to the absorption range of 390–425 nm. We studied the impact of temperature on the luminescence intensity and lifetime of the films excited by visible light. The original result of this work is high thermal sensitivity (18.97 μs/K) of the produced films excited by visible light (400 nm). It was demonstrated that the studied temperature sensitive materials can be excited by inexpensive sources of visible light, which can contribute to enhancing the photostability of a sensing element and reducing the overall cost of temperature measurement devices. The results of this work can be useful for development of new temperature sensors for applications in biology, medicine, industry, and fundamental research.

## 2 Experimental

### 2.1 Materials and methods

Europium (III) chloride hexahydrate (EuCl_3_·6H_2_O) (99.9%), 1,10-Phenanthroline (99%) and the solvents were purchased from Sigma-Aldrich (Sigma-Aldrich, Germany). CHN analysis was carried out on Delta V Plus Thermo Fisher Scientific isotopic mass spectrometer. The phase transition temperatures of the Eu(III) complex were measured for its powder using Olympus BX-51 polarized light microscope equipped with high precision Lincam heating system. Thermogravimetric analysis (TGA) of the studied samples were determined on TGA/DSC 1 Star system differential scanning calorimeter by Mettler Toledo in the heating/cooling mode and scanning rates of 10 K/min in sealed aluminum containers. Absorption and transmission spectra were recorded on Lambda-35 Perkin–Elmer UV/Vis spectrophotometer. Luminescent properties were characterized by Cary Eclipse Varian spectrofluorimeter. Size characterization of the aggregates represented by Ln (III) complexes in the films was performed on Carl Zeiss Evo scanning electron microscope.

### 2.2 Synthesis of Eu(III) complex

Synthesis of *tris[1-(4-(4-propylcyclohexyl)phenyl)octane-1,3-dione]-[1,10-phenanthroline]europium* was performed according to the method described in literature (Galyametdinov et al., 2022; Knyazev et al., 2022b; Lapaev et al., 2018b). Alcohol solution of 0.040 g (0.11 mmol) EuCl_3_·6H_2_O was added slowly dropwise to hot alcohol solution of 0.113 g (0.33 mmol) β-diketone, 0.020 g (0.11 mmol) 1,10-phenanthroline and 0.018 g (0.32 mmol) KOH and agitated constantly. The emerging light-yellow precipitate was separated by hot filtration, washed by hot alcohol and dried in vacuum. The synthesized product was washed with toluene. The resulting solution was vaporized in vacuum to obtain the dried final product.

The structure of the synthesized C_81_H_107_EuN_2_O_6_ compound was confirmed by elemental analysis and mass spectrometry. The melting temperature Т_melt_ = 130 °С. Calculated (%): C, 71.60; H, 7.99; N, 2.04; O, 7.22, Eu, 11.05. Found (%):C 71.11; H, 8.25; N 2.00; O, 7.38, Eu, 11.20. ESI-MS (m/z) 1380 (M + Na)^+^.

### 2.3 Fabrication of film materials

Nanoscale film materials were fabricated by performing spin-coating of Eu(III) complexes from their toluene solutions with the concentration of 1 × 10^−3^ mol/L. Microscale films were fabricated by two methods: vitrifying the melt of Eu(III) powder between two quartz substrates and evaporating solvent from solution at normal conditions.

### 2.4 Quantum yields

The relative luminescence quantum yields φ of the films of the complexes were calculated by using Equation 1 (Bazan et al., 1996):

$$\varphi =\varphi_{std}\times \left[\frac{A_{std}\left(\lambda_{std}\right)}{A_{unk}\left(\lambda_{unk}\right)}\right]\times \left[\frac{I_{std}\left(\lambda_{std}\right)}{I_{unk}\left(\lambda_{unk}\right)}\right]\times \left[\frac{D_{unk}}{D_{std}}\right]$$ (1)

The subscripts *std* and *unk* indicate the standard and unknown samples, respectively; *A(λ)* corresponds to the absorbance of the films at the excitation wavelength *λ*; *I(λ)* is the intensity of the exciting beam (assumed to be equal for both measurements); *D* is the integrated luminescence spectrum. The standard fluorophore for the measurements was a thin film of poly (methyl methacrylate) (PMMA) co-doped with 1% Eu (tta)_3_(H_2_O)_2_ with *φ*_*std*_ = 45% (Kai et al., 2011).

### 2.5 Calculation of the thermal sensitivity

The absolute thermal sensitivity (S^a^) was calculated by Equation 2 (Collins et al., 1998):

$$S^{a}=\frac{\partial \Delta }{\partial T}$$ (2)

where Δ is the luminescence intensity or lifetime.

The relative thermal sensitivity (S^r^) was calculated by Equation 3 (Collins et al., 1998):

$$S^{r}=\frac{|\partial \Delta |}{\partial T\times \;\Delta }\times 100\%$$ (3)

## 3 Results and discussion

**FIGURE 1:**
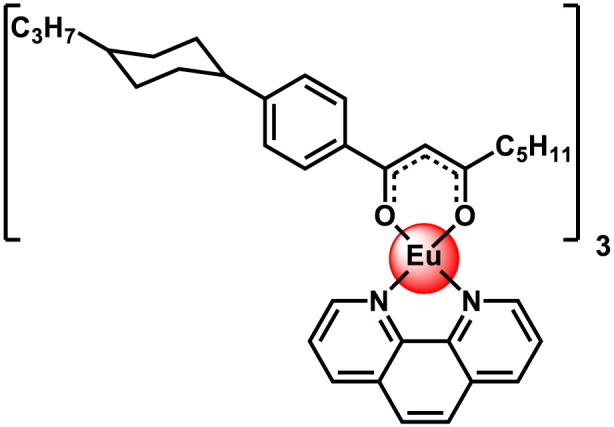
Structure of the β-diketonate complex of europium (III).

In this work, we synthesized the Eu(III) complex with the structure shown in Figure 1. Its composition and structure were confirmed by X-ray diffraction (Knyazev et al., 2025), mass spectrometry, and elemental analysis. A characteristic feature of the proposed β-diketonate Ln (III)complexes is their anisometric molecular structure with cyclohexane and long hydrocarbon substituents at the edges of their molecules (Figure 1). This structure hinders crystallization, allows for a substantial lowering of the softening temperature, and avoids decomposition processes during melting, which occur with most known analogues. According to differential scanning calorimetry and polarized optical microscopy data these compounds can vitrify (Knyazev et al., 2025). The Eu(III) complex is thermostable in the range of 20–200 °С as shown by TGA (Supplementary Figure S1). With such properties, it is possible to produce film materials from the powder of the complex, which can be used as luminescent temperature sensors.

**FIGURE 2:**
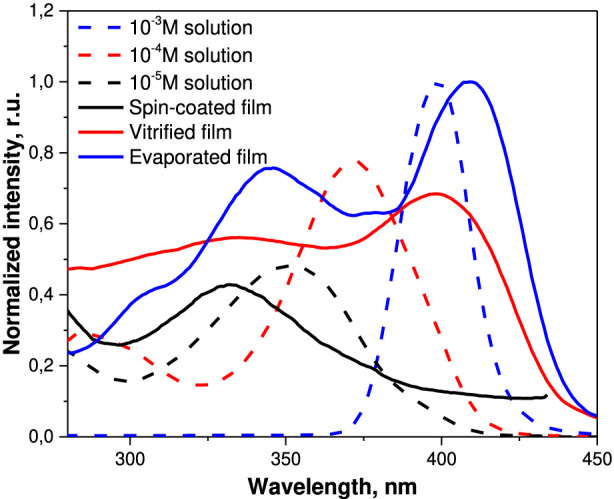
Excitation spectra of solutions and films of the Eu(III) complex at λ_em_ = 613 nm.

In addition, these complexes dissolve well in nonpolar and low-polar organic solvents (Knyazev et al., 2015; Knyazev et al., 2023; Lapaev et al., 2018a). Excitation of hexane solution (10^–5^ mol/L) of the synthesized Eu(III) complex corresponds to π- π*-transitions of β-diketones. They make the major contribution to the transfer of energy to Eu^3+^ ions (the “antenna effect”). As shown in the example of solutions in hexane the excitation spectra of the Eu(III) complex demonstrated a shift of their maximums to a longer wave area (Figure 2), which may indicate formation of J-aggregates as it was previously shown for BODIPY and its derivatives (Cheng et al., 2023; Choi et al., 2014; Kim et al., 2020).

The synthesized Eu(III) complex was used to fabricate films by three different methods: spin-coating, vaporization of solvent, and vitrification from melt. Considerable differences were observed in the supramolecular organization and optical properties of the synthesized films. The excitation spectra (Figure 2) showed that the films fabricated by vitrification and solvent evaporation contain aggregates similar to the ones in concentrated solutions. In the spin-coated films, virtually no aggregation occurred and the resulting spectra are similar to the ones of diluted solutions.

The authors in (Knyazev et al., 2025) supposed that there are two reasons behind this effect: rapid removal of excess solvent by spin-coating (Birnie, 2013; Mendhe, 2023) disrupts intermolecular interactions and prevents particles from agglomeration. Another reason can be removal of large and heavy aggregates from the surface of the film by centrifugal force (Das et al., 2020; Kotsuki et al., 2014).

**FIGURE 3:**
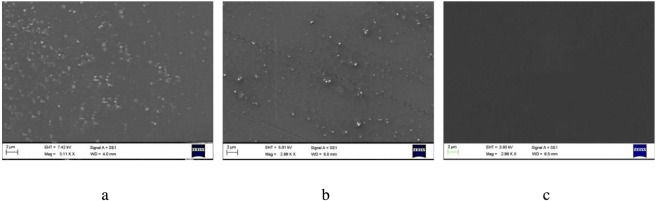
SEM images of the surface of films deposited by glass transition from the melt **(a)**, solvent evaporation **(b)** and spin-coating **(c)**.

The absence of crystallization in the obtained films was shown by polarized optical microscopy (Supplementary Figure S2). Scanning electron microscopy studies revealed that amorphous aggregates (approximately 300–700 nm in size) are present in Eu(III) complex films obtained by glass transition from the melt and solvent evaporation (Figures 3a,b; Supplementary Figure S3). In the spin-coated films obtained from concentrated solutions, aggregates are not observed (Figure 3c).

The obtained films were also studied by the small-angle X-Ray scattering (SAXS) method (Knyazev et al., 2025), which allows one to determine the type of intermolecular interactions for aggregates with size up to 300 Å. Considering that there are no active functional groups in the ligand molecules, in early work it was found that the main intermolecular interactions in the crystal of the complex are C-H … O hydrogen bonds and multiple π … π and C-H … π contacts. Molecules align in a “head-to-tail” or slipped and form J-aggregates.

The authors suggest that the formation of supramolecular structures due to J-aggregation leads to a change in the triplet levels of organic ligands and, as a consequence, the excitation bands as is known for BODYPY (Cheng et al., 2023; Choi et al., 2014; Kim et al., 2020) As shown by the authors earlier, using DLS data, in concentrated solutions, almost all molecules of the complex form solvated aggregates (Knyazev et al., 2025), which is the reason for the bathochromic shift of the excitation maximum. The films contain aggregates of the complex molecule with sizes ranging from several (according to SAXS data) to hundreds (according to SEM data) nanometers. Therefore, several maxima are observed in the excitation spectra of the films (Figure 2). In microscale films, large aggregates prevail, while in nanoscale films they are almost absent. In the obtained films, the formation of J-aggregates shifts the absorption and excitation spectra toward longer wavelengths.

**FIGURE 4:**
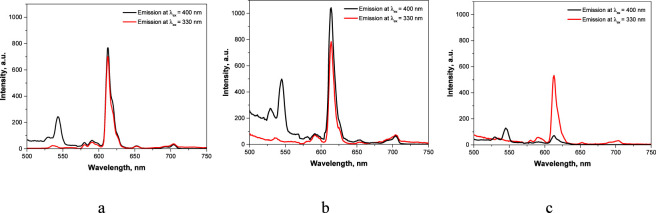
Luminescence spectra of Eu(III) complex films: vitrified **(a)**, evaporated **(b)** and spin-coated **(c)** (at λ_ex_ = 330 and 400 nm).

The luminescent properties of films of Eu(III) complexes were compared under excitation at 330 nm and 400 nm (Figure 4). As shown in Figure 4a, the luminescence spectra of the vitrified films obtained under both excitation wavelengths are nearly identical. This observation is further supported by the calculated relative quantum yields (Equation 1), which were 30.1% at 330 nm and 30.4% at 400 nm. These results demonstrate the possibility of using low-cost visible light sources emitting in the 390–425 nm range. A similar situation is observed for the evaporated film (Figure 4b), where large aggregates prevail. The spin-coated film exhibits weak luminescence under excitation at 400 nm due to the absence of J-aggregation (Figure 4c), which is consistent with the excitation spectra (Figure 2).

Using of UV light sources for luminescence excitation is impractical due to their high cost and the risk of photodegradation of phosphors. Microscale films, which were vitrified between quartz substrates, were found to be photostable in UV (365 nm) and visible light irradiation conditions because the phosphor was isolated from the environment. The films, which were produced by solvent evaporation and spin-coating show losses of more than 30% of luminescence intensity after prolonged UV (365 nm) excitation (Knyazev et al., 2025) and therefore their study as temperature sensors was not of interest.

**FIGURE 5:**
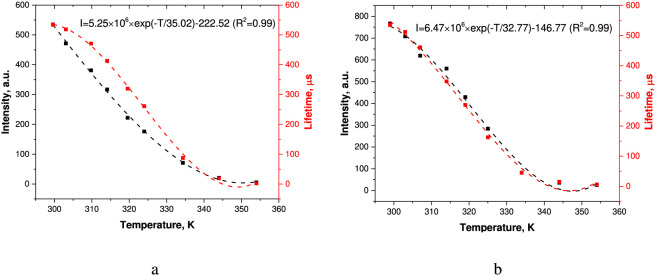
Temperature dependences of the luminescence intensity and lifetime at λ_em_ = 613 nm, λ_ex_ = 330 nm **(a)** and 400 nm **(b)**.

According to the literature data is well known that the luminescence intensity of thermosensitive films depends strongly on characteristics of a sample and measurement conditions (Brites et al., 2016; Wang et al., 2013). In addition, it is rather challenging to consider degradation of films under irradiation and avoid the resulting considerable temperature measurement errors (Borisov and Klimant, 2008; Borisov and Klimant, 2012; Borisov and Wolfbeis, 2006). The emission intensity of a temperature sensor can depend considerably on the refraction index of a medium (for example, contamination of the surface of a temperature sensor by water or other liquids) or the presence of biological or chemical substances. In contrast, the luminescence lifetime does not depend on these factors (Brites et al., 2016; Wang et al., 2013). Therefore, this parameter is often used as a more reliable and accurate temperature indicator. The luminescence intensity depends substantially on experimental conditions and characteristics of samples (Lapaev et al., 2018b). Another contribution to measurement errors is provided by degradation of materials. In its turn, the luminescence lifetime does not depend on these factors and offers a more accurate method of temperature measurement (Dramićanin, 2020). Thus, the next stage of this work focused on studying the impact of temperature on the luminescent properties of vitrified films made from the Eu(III) complexes. Figure 5 shows the effect of temperature on the luminescence intensity of the films in the 298–353 K range at the excitation wavelength λ_ex_ = 330 and 400 nm and the emission wavelengths λ_em_ = 613 nm.

An increase in the temperature leads to a nonlinear decrease in the luminescence intensity and lifetime of the Eu(III) complexes at λ_ex_ = 330 nm and 400 nm and λ_em_ = 613 nm (Figure 5; Supplementary Figure S4; Supplementary Table S1) The temperature behavior of the luminescence intensity of a microscale film is adequately described by an exponential function with the correlation coefficients R^2^ > 0.99 (for λ_ex_ = 613 nm). A decrease in the luminescence intensity and lifetime of Ln (III) compounds at higher temperatures is known to be caused by its temperature quenching, which is initiated by nonradiative relaxation processes (Lapaev et al., 2019).

**FIGURE 6:**
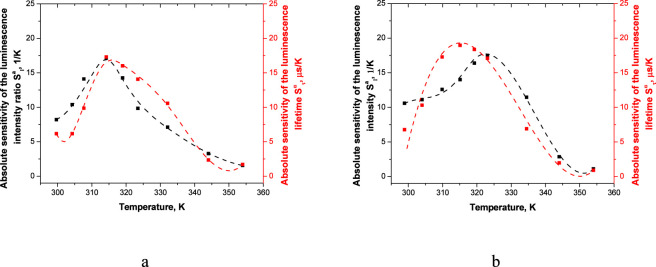
Absolute sensitivity of the luminescence lifetime (red curve) and luminescence intensity (black curve) of the vitrified films at λ_ex_ = 330 nm **(a)** and 400 nm **(b)**.

**FIGURE 7:**
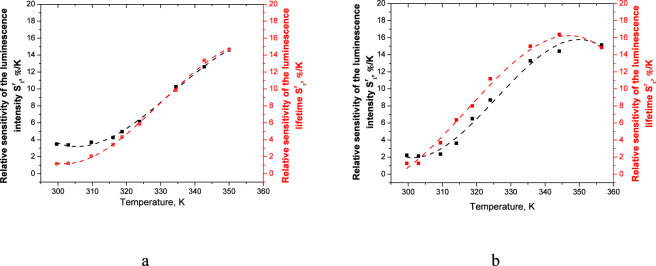
Relative sensitivity of the luminescence lifetime (red curve) and luminescence intensity (black curve) of the vitrified films at λ_ex_ = 330 nm **(a)** and 400 nm **(b)**.

Figures 6, 7 represent absolute and relative sensitivities of the luminescence lifetimes and intensities (Equation 2) of microscale (Equation 3) films based on Eu(III) complexes.

The vitrified film demonstrates a high absolute temperature sensitivity of the luminescence lifetime S^a^_τ_. When excited at a wavelength of λ_ex_ = 330 nm the S^a^_τ_ value varies from 6.11 μs/K at 298 K to 1.68 μs/K at 353 К with the max sensitivity of 17.30 μs/K at 313 K (Figure 6a). When excited at 400 nm values of S^a^τ change from 6.77 μs/K at 298 K to 0.9 μs/K at 353 К with the max sensitivity of 18.97 μs/K at 313 К. This corresponds to the best examples of lanthanide-containing thermosensors and significantly exceeds parameters of known visible-light-excited analogues. (Figure 6b) (Table 1). The vitrified film also demonstrates a high absolute temperature sensitivity of the luminescence intensity S^a^_I_. The max sensitivity S^a^_I_ value is 17.15 1/K at 313 K (excitation at a wavelength of 330 nm), (Figure 6a), and 17.51 1/K at 323 K (excitation at a wavelength of 400 nm) (Figure 6b) in the temperature range 298–353 K respectively.

**TABLE 1:** The known visible-light-excited thermosensitive materials based on Ln (III) complexes.

Sensor film	λ_max_, нм	Operating temperature range, K	Quenching time range, µs	S^a^_I_ 1/K	S^r^_I_ %/K	S^a^_τ_ µs/K	S^r^_τ_ %/K	Degradation (%/h)	References
Eu(CPDK_3-5_)_3_Phen	330	298–353	534–2	17.15	14.61	17.30	14.67	No	This work
Eu(CPDK_3-5_)_3_Phen	400	298–353	535–6	17.51	15.15	18.97	16.39	No	This work
(Me_2_NH_2_)_3_ [Ln_3_(FDC)_4_(NO_3_)_4_] ·4H_2_O	450	12–320	​	-	2.7 (170K), 0.33 (300 K)	-	-	​	Li et al. (2016)
Eu (tta)_3_dpbt in PVMK	411	274–339	517–200	-	-	4.9	−0.94	7	Borisov and Wolfbeis (2006)
Eu (tta)_3_DEADIT	416	274–323	360–130	-	-	−4.7	−1.3	20	Borisov and Klimant (2008)
Eu (DTP)_3_	412	283–323	380–200	-	-	−4.5	−1.2	17	Ondrus et al. (2015)
Eu (TTA)_3_	395	273–363	350–128	-	-	−2.5	−0.7	-	Borisov and Klimant (2012)

The vitrified film also demonstrates high values of the relative sensitivity of the luminescence lifetime S^r^_τ_ in the temperature range 298–353 K. The max sensitivity 14.67%/K at 353K excitation at 330 nm (Figure 7a) and 16.39%/K at 343K excitation at a wavelength of 400 nm (Figure 7b) respectively. The max sensitivity value of luminescence S^r^_I_ is 14.61%/K at 353 K excitation at 330 nm (Figure 7a) and 15.15%/K at 353 K excitation at a wavelength of 400 nm (Figure 7b) respectively. The operating characteristics of the produced thermosensitive elements and known analogues are summarized in Table 1. Obtained sensor exceeds considerably the existing performance of known visible-light-excited thermosensitive materials based on Ln (III) complexes (Borisov and Klimant, 2008; Borisov and Klimant, 2012; Borisov and Wolfbeis, 2006; Li et al., 2016; Ondrus et al., 2015).

Table 1 shows that the sensitivities of the vitrified films evaluated by increasing the wavelength from 330 nm to 400 nm do not reduce and even show a 10% increase. This eliminates, the need for using UV light sources for excitation of the complexes and makes it possible to utilize less inexpensive visible-light sources with the wavelength of 400 nm.

**FIGURE 8:**
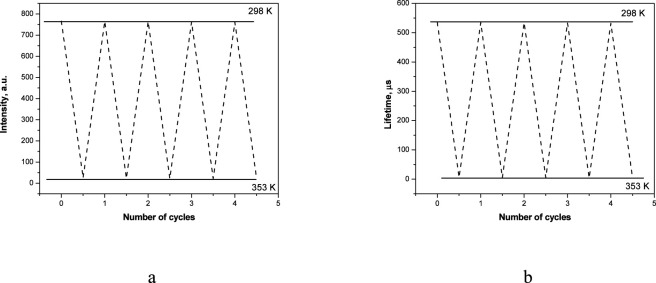
Changes in luminescence intensity **(a)** and lifetime **(b)** in five consecutive heating (353 K) and cooling (298 K) cycles.

From an application point of view, an important property of film materials is reversibility of their luminescence intensity and lifetime, which makes the basis for their prospective applications as reusable sensors for luminescent thermometers. The thermal stability of the produced microscale films was evaluated by monitoring the intensity of the emission bands at the wavelength of 613 nm (Eu^3+^) and the luminescence lifetime of europium (Figure 8a) in consecutive cycles of heating to 353 K and cooling down to 298 K. The vitrified film excited at 400 nm was used as an example.

According to the experimental data, the films withstand at least five consecutive cycles and the intensity losses were relatively small (less than 5%) (Figure 8a). The thermal stability of the luminescence lifetime values of the vitrified films was evaluated in a similar way. After multiple heating and cooling cycles, the lifetime remained virtually the same (Figure 8b). This important experimental fact confirms the complete reversibility of the luminescence lifetime of the Eu(III) ions depending on temperature.

Due to the properties described above, the studied vitrified films can be a promising material for producing inexpensive and reusable visible-light-excited temperature sensors for the range of 298–353 K.

## 4 Conclusion

In this work, we synthesized an anisometric Eu(III) complex. Its structural features allowed to fabricate photostable microscale films with a uniform structure and tailored optical properties. J-aggregates in the produced films shift the absorption and excitation spectra to a longer wave area. The luminescence spectra of the microscale film excited at 330 nm and 400 nm are virtually identical. It was also confirmed by the calculated relative quantum yields of luminescence after excitation at 330 nm and 400 nm, which were found to be 30.1% and 30.4%, respectively. The effect of temperature on luminescent properties was studied upon excitation at a wavelength of 400 nm. The sensitivity values of the luminescence intensity S^a^_I_ and lifetime S^a^_τ_ when excited at a wavelength λ_ex_ = 400 nm are 17.51 1/K and 18.97 μs/K, which significantly exceeds the existing performance of known visible-light excited thermosensitive materials based on Ln (III) complexes and corresponds to the best analogues excited by UV light. The obtained materials can be used in the production of inexpensive, reusable visible-light-excited temperature sensors sensitive in the range of 298–353 K for applications in biology, medicine, industry, and fundamental research.

## Data Availability

The original contributions presented in the study are included in the article/Supplementary Material, further inquiries can be directed to the corresponding author.
