# Supplementary material for: Lanthanide-containing visible-light-excited thermosensitive luminescent films
Source: Front Chem. 2026 Mar 25;14:1786361. doi: 10.3389/fchem.2026.1786361 (PMC13057526; doi:10.3389/fchem.2026.1786361)
Supplement: Supplementary file 1 [file DataSheet1.pdf]

Supplementary Material

1 Supplementary Figures and Tables

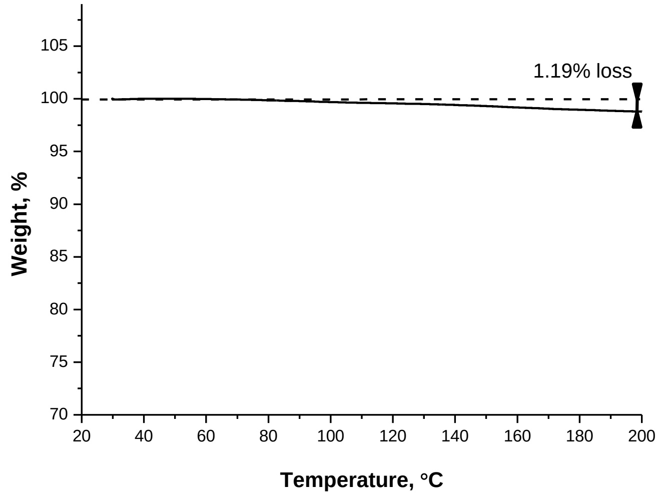

Supplementary Figure 1. TGA curve of the Eu(III) complex.

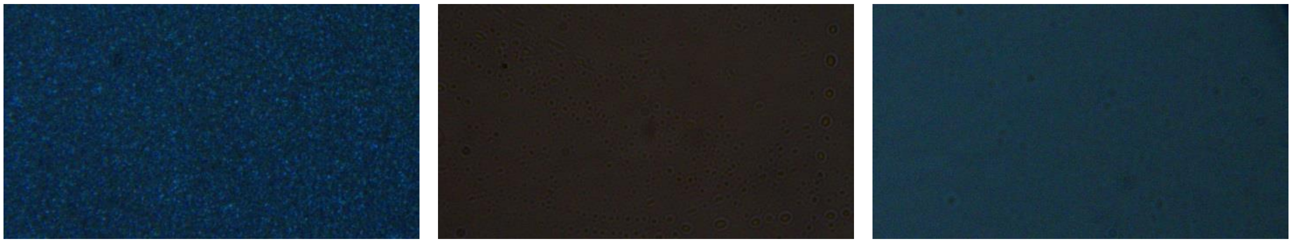

a b c

Supplementary Figure 2. Micrograph of the surface of the films deposited by glass transition from the melt (a), solvent evaporation (b) and spin-coating(c) in the crossed polarizers mode at 1000× magnification.

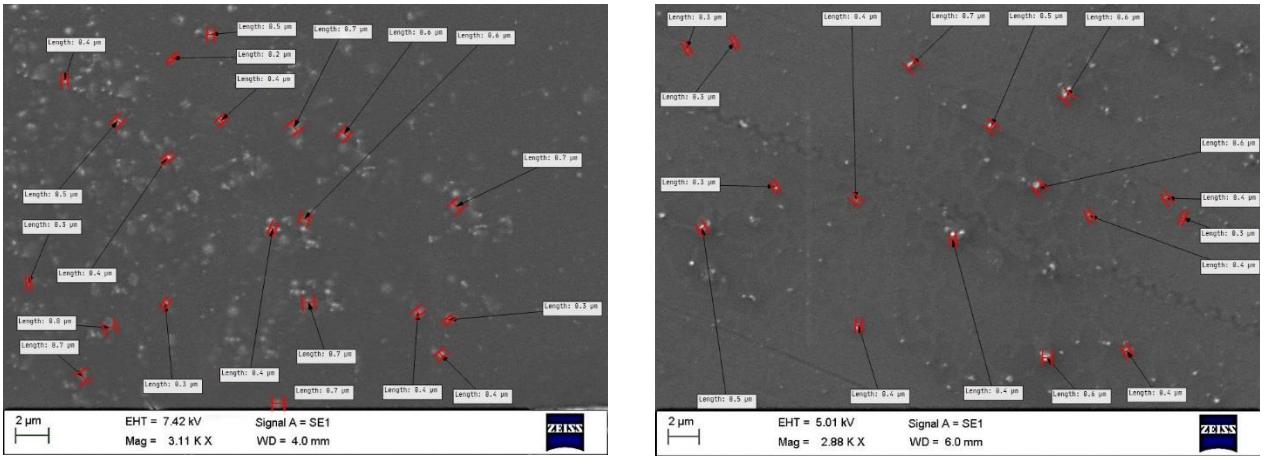

a b

Supplementary Figure 3. SEM images of the surface of films deposited by glass transition from the melt (a) and solvent evaporation (b).

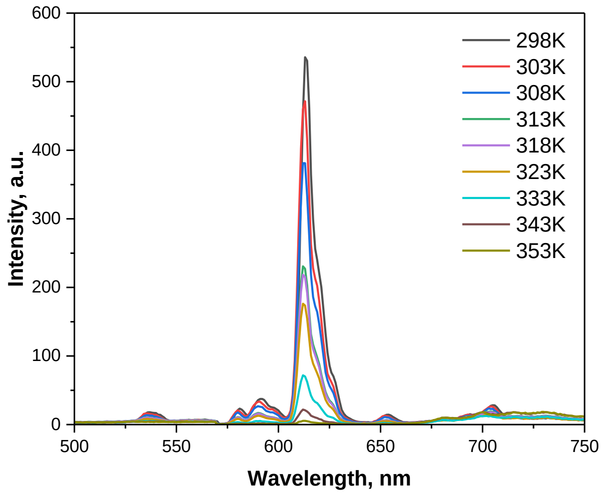

a

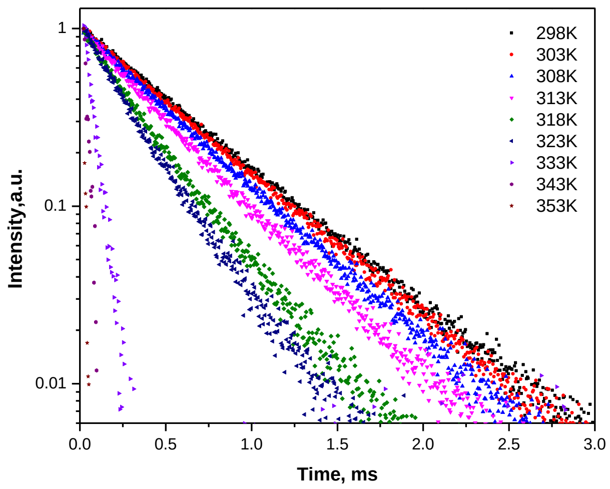

b

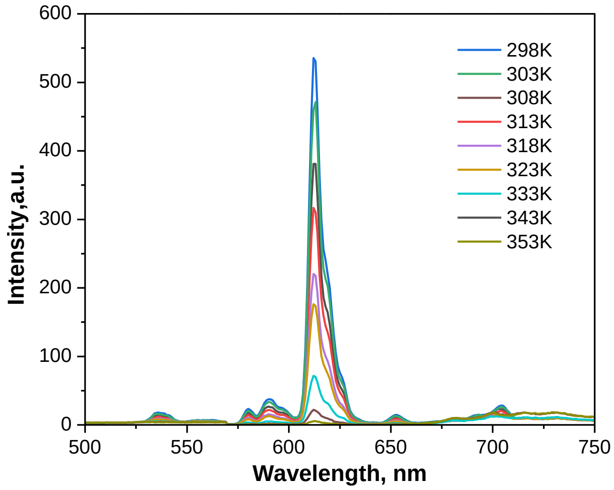

c

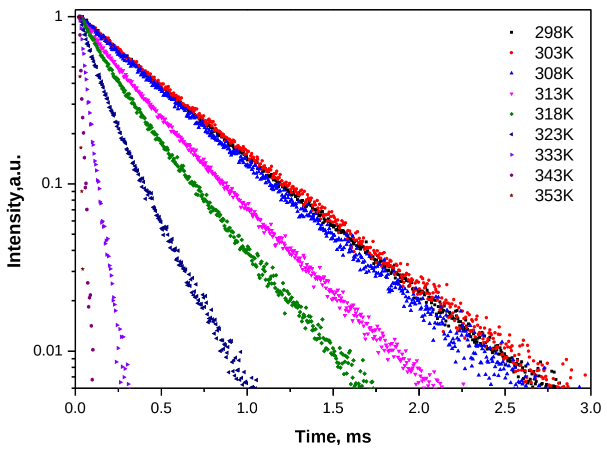

d

Supplementary Figure 4. Luminescence spectra (a,c) and lifetime (b,d) at different temperature upon excitation at 330 nm (a,b) and 400 nm (c,d).

Supplementary Table 1. Luminescence lifetime dependence on temperature upon excitation at 330 nm and 400 nm

Temperature, K	Lifetime, µs	
	λ_{ex}=330 nm	λ_{ex}=400 nm
298	534	535
303	518	512
308	471	461
313	413	348
318	320	270
323	261	162
333	87	45
343	19	15
353	2	6
